# YOLO-PLNet: a lightweight real-time detection model for peanut leaf diseases based on edge deployment

**DOI:** 10.3389/fpls.2025.1707501

**Published:** 2025-11-18

**Authors:** Jinti Sun, Zhihui Feng, Jiaqi Han, Fulei Xu, Hui Zhang, Yufeng Guo

**Affiliations:** College of Information and Management Science, Henan Agricultural University, Zhengzhou, China

**Keywords:** peanut leaf disease, real-time detection, edge computing, Jetson platform, lightweight model

## Abstract

As an important economic crop, peanut is frequently affected by leaf diseases during its growth period, which severely threaten its yield and quality. Therefore, early and accurate disease detection is critical. However, existing lightweight deep learning methods often struggle to balance model size, real-time detection accuracy, and edge device deployment, limiting their widespread application in large-scale agricultural scenarios. This study proposes a lightweight real-time detection model, YOLO-PLNet, designed for edge deployment. The model is based on YOLO11n, with lightweight improvements to the backbone network and Neck structure. It introduces a Lightweight Attention-Enhanced (LAE) convolution module to reduce computational overhead and incorporates a Channel-Spatial Attention Mechanism (CBAM) to enhance feature representation for small lesions and edge-blurred targets. Additionally, the detection head adopts an Asymptotic Feature Pyramid Network (AFPN), leveraging staged cross-level fusion to improve detection performance across multiple scales. These improvements significantly enhance the detection accuracy of peanut leaf diseases under complex backgrounds while improving adaptability for edge device deployment. Experimental results show that YOLO-PLNet achieves a parameter count, computational complexity, and model size of 2.13M, 5.4G, and 4.51MB, respectively, representing reductions of 18.07%, 16.92%, and 15.70% compared to the baseline YOLO11n. The mAP@0.5 and mAP@0.5:0.95 reach 98.1% and 94.7%, respectively, improving by 1.4% and 1.7% over YOLO11n. When deployed on the Jetson Orin NX platform with real-time video input from a CSI camera, the model achieves a latency of 19.1 ms and 28.2 FPS at FP16 precision. At INT8 precision, latency is reduced to 11.8 ms, with real-time detection speed increasing to 41.3 FPS, while GPU usage and power consumption are significantly reduced with only a slight decrease in detection accuracy. In summary, YOLO-PLNet achieves high detection accuracy and robust edge deployment performance, providing an efficient and feasible solution for intelligent monitoring of multiple categories of peanut leaf diseases.

## 1 Introduction

Peanut (Arachis hypogaea L.) (Asif et al., 2025) is a critical oilseed and economic crop, playing a pivotal role in agricultural production. However, peanut plants are highly susceptible to various diseases, such as leaf spot and rust, during their growth, which can lead to severe defoliation and yield losse (Duffeck et al., 2025). Currently, disease management in peanuts faces two major challenges: firstly, the long-term reliance on chemical control has resulted in increased pathogen resistance, pesticide residue accumulation, and growing concerns regarding environmental and food safety issues (Munir et al., 2024); secondly, traditional diagnostic methods, such as field visual inspection and laboratory testing, are inefficient, costly, and time-consuming, failing to meet the demands of modern agriculture for early warning and large-scale, real-time disease monitoring (Reddy et al., 2024).

With the advancement of smart agriculture, plant disease detection technology based on computer vision has emerged as a mainstream solution due to its non-contact, automated, and highly efficient characteristics (Ghazal et al., 2024). Researchers have extensively explored the application of deep learning models in crop disease identification. For instance, Miao et al. proposed the SerpensGate-YOLOv8 model, which incorporates DySnakeConv, SPPELAN, and STA attention mechanisms, achieving a precision of 71.9% on the publicly available PlantDoc dataset t (Miao et al., 2025). Similarly, Ayon et al. conducted a systematic comparison of four deep learning models—VGG16, ViT, EfficientNetB3, and Xception—using a tea leaf image dataset, validating the effectiveness of Transformer architectures and traditional CNNs in tea disease identification (Ayon et al., 2024).

However, such models often rely on high-performance servers or desktop-grade GPU platforms, making stable operation on resource-constrained embedded edge devices challenging due to limitations in storage and power consumption (Cajas Ordóñez et al., 2025). In peanut cultivation scenarios, diseases are widely distributed with high density, and single-frame image detection cannot adequately cover continuous areas. Consequently, real-time video detection using low-power, compact edge AI devices has emerged as a promising development direction. By deploying inference on edge platforms with computational capabilities (e.g., Jetson Orin NX, Jetson Orin Nano), on-site inference in the field can be achieved, significantly reducing uplink bandwidth and transmission latency while ensuring continuous monitoring under weak network conditions. Additionally, these devices can synergize with field operation units such as drones and autonomous agricultural machinery, reducing labor costs and enhancing the efficiency of pesticide application or irrigation.

To address these challenges, researchers have increasingly focused on designing lightweight network architectures, such as YOLO, MobileNet, ShuffleNet, and EfficientNet, to enable efficient deployment of disease detection models on edge AI devices. Zhang et al. proposed the GVC-YOLO model, tailored for real-time detection of cotton aphid leaf diseases on edge devices. Built upon YOLOv8n, the model integrates GSConv, VoVGSCSP, SimSPPF, and a coordinate attention mechanism, achieving a compact model size of 5.4 MB while maintaining a detection accuracy of 97.9% and a real-time inference speed of 48 FPS on the Jetson Xavier NX platform (Zhang et al., 2024). Similarly, Xie et al. introduced YOLOv5s-BiPCNeXt, which incorporates a MobileNeXt backbone, C3-BiPC structure, EMA attention mechanism, and CARAFE upsampling operation. This model achieves high-precision eggplant disease detection at 26 FPS on the Jetson Orin Nano, effectively balancing deployment efficiency and recognition performance (Xie et al., 2024b). Additionally, a comprehensive review systematically outlined key technologies and architectures for edge AI and IoT in real-time crop disease detection, categorizing them into four types: edge sensing and processing frameworks, lightweight models for resource-constrained devices, data-driven approaches, and communication protocols tailored for agricultural scenarios. The review highlighted that CNNs based on MobileNet/EfficientNet, combined with techniques such as pruning and quantization, have enabled effective detection on hardware like Raspberry Pi and Jetson Nano (Madiwal et al., 2025).

Building on these research directions, we developed an efficient peanut leaf disease detection model, YOLO-PLnet, and deployed it on the Jetson Orin NX edge device. Based on YOLO11n, we integrated the Lightweight Attention-Enhanced convolution module, which enhances the model’s ability to represent edge details and target regions. Additionally, we leveraged the cross-stage feature fusion capabilities of the Asymptotic Feature Pyramid Network to improve detection accuracy. During the deployment phase, we employed TensorRT for precision optimization and inference acceleration, deploying the model on a Jetson Orin NX edge platform equipped with an industrial-grade CSI camera. Real-time detection was performed using field-captured video streams, and the model’s performance was systematically evaluated in terms of latency, frame rate, memory usage, and power consumption under FP16, INT8, and multi-resolution settings. Overall, this study provides a viable technical solution for the precise identification and intelligent management of peanut leaf diseases.

## 2 Materials and methods

### 2.1 Data acquisition and description

Deep learning has demonstrated significant potential in plant disease detection, but its performance heavily relies on high-quality and large-scale datasets. Existing studies, particularly those focused on peanut leaf diseases, often utilize public datasets that cover only a limited range of disease categories, such as early leaf spot and rust, resulting in incomplete category coverage and dataset imbalance. This leads to the neglect of critical conditions, such as nutrient deficiencies (Ridoy et al., 2024). To address this issue, we constructed a novel peanut leaf disease dataset encompassing six major disease categories, significantly enhancing the dataset’s comprehensiveness and representativeness. This provides a richer and more balanced sample set for model training.

Data collection was conducted from late June to mid-September 2024 in over 20 representative peanut fields in Zhengzhou City, Henan Province, China, covering the typical disease stages of major leaf diseases throughout the peanut growth cycle. Image data were primarily captured using a Fuji FinePix S4500 digital camera, with an image resolution of 2017×2155 pixels, and saved in JPG format. The distance between the camera and the leaves was controlled within 20–35 cm. Image acquisition was mainly performed at three time slots—7:00 AM, 1:00 PM, and 5:00 PM—to simulate different lighting conditions in natural environments.

**Figure 1:**
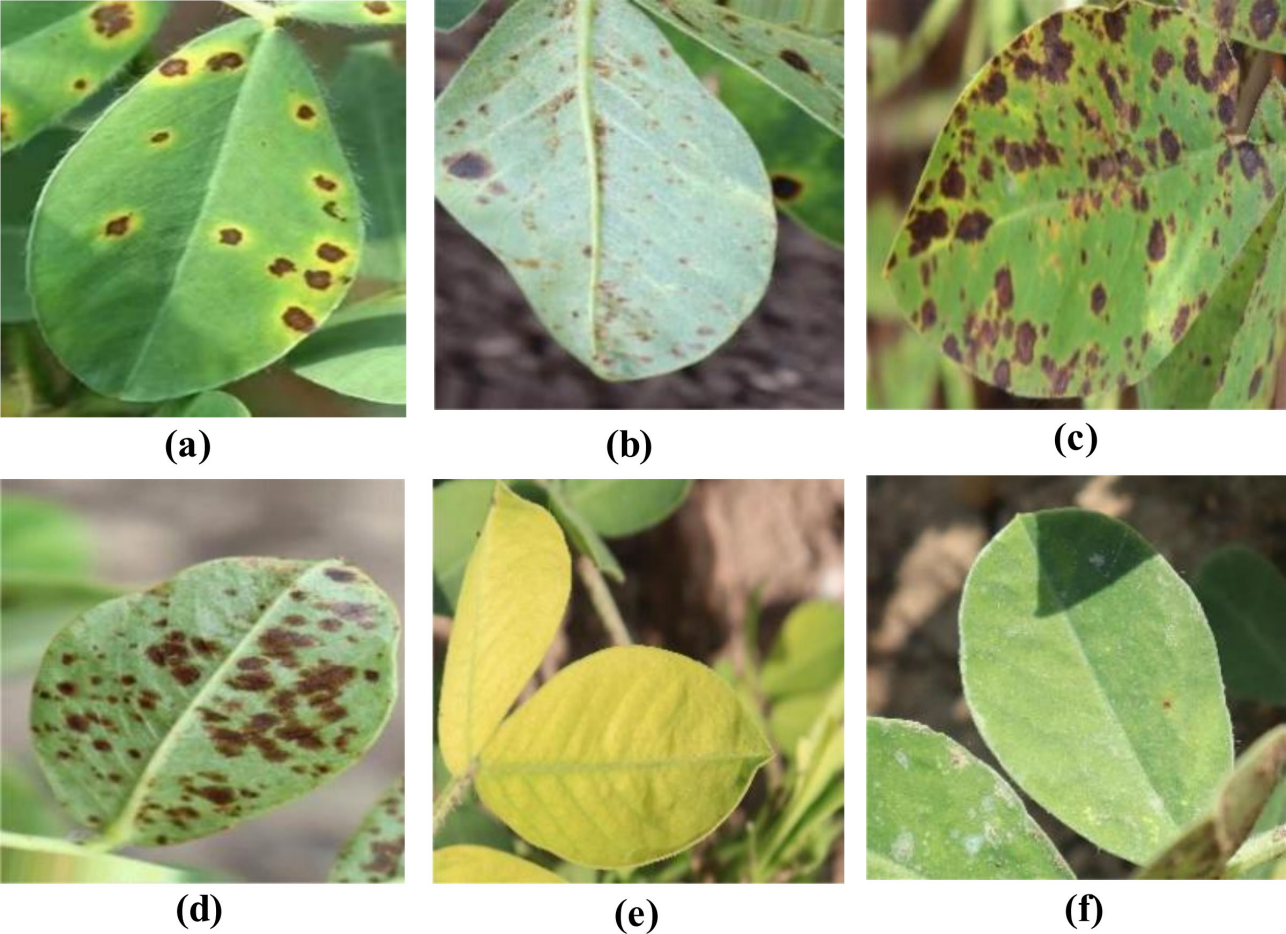
Sample images from the peanut leaf disease dataset. **(A)** Early Leaf Spot. **(B)** Early Rust. **(C)** Late-leaf-spot. **(D)** Rust.**(E)** Nutrition-Deficiency. **(F)** Healthy.

During the data collection process, various shooting angles (including eye-level, top-down, and side views) and diverse natural lighting conditions (such as sunny, cloudy, and backlit scenarios) were fully considered to capture typical field scene characteristics. The dataset covers all stages of disease progression, from initial onset to severe development. The dataset comprises six categories: early leaf spot disease, early rust disease, late leaf spot disease, late rust disease, nutrient deficiency, and healthy leaves. Through a rigorous image quality screening process, a total of 2,132 high-quality original image samples were retained, providing a solid data foundation for subsequent model training and evaluation. The dataset classification standards are presented in **Table 1**, and samples from the peanut leaf disease dataset are shown in **Figure 1**.

**Table 1:** Classification criteria for the peanut leaf disease dataset.

Label	Name	Description
0	Early Leaf Spot	Small, round, reddish-brown lesions appearing primarily on the lower leaf surface
1	Early Rust	Tiny orange or yellowish pustules on the upper leaf surface during early infection
2	Late-leaf-spot	Large dark brown or black lesions with concentric rings, often coalescing on leaves
3	Nutrition-Deficiency	General yellowing, stunted growth, or vein chlorosis not associated with pathogens
4	Rust	Numerous rust-colored pustules forming dense clusters on both leaf surfaces
5	Healthy	Leaves with uniform green color, intact texture, and no visible disease symptoms

### 2.2 Data analysis and preprocessing

To achieve precise detection and identification of peanut leaf disease regions, under the guidance of plant protection experts, we employed the LabelImg (Pande et al., 2022) tool for manual annotation of disease targets in the collected images. Annotations were based on the minimum bounding rectangle for each target, ensuring that the bounding box closely fits the disease region to minimize background interference, thereby enhancing the localization accuracy of subsequent models.

**Figure 2:**
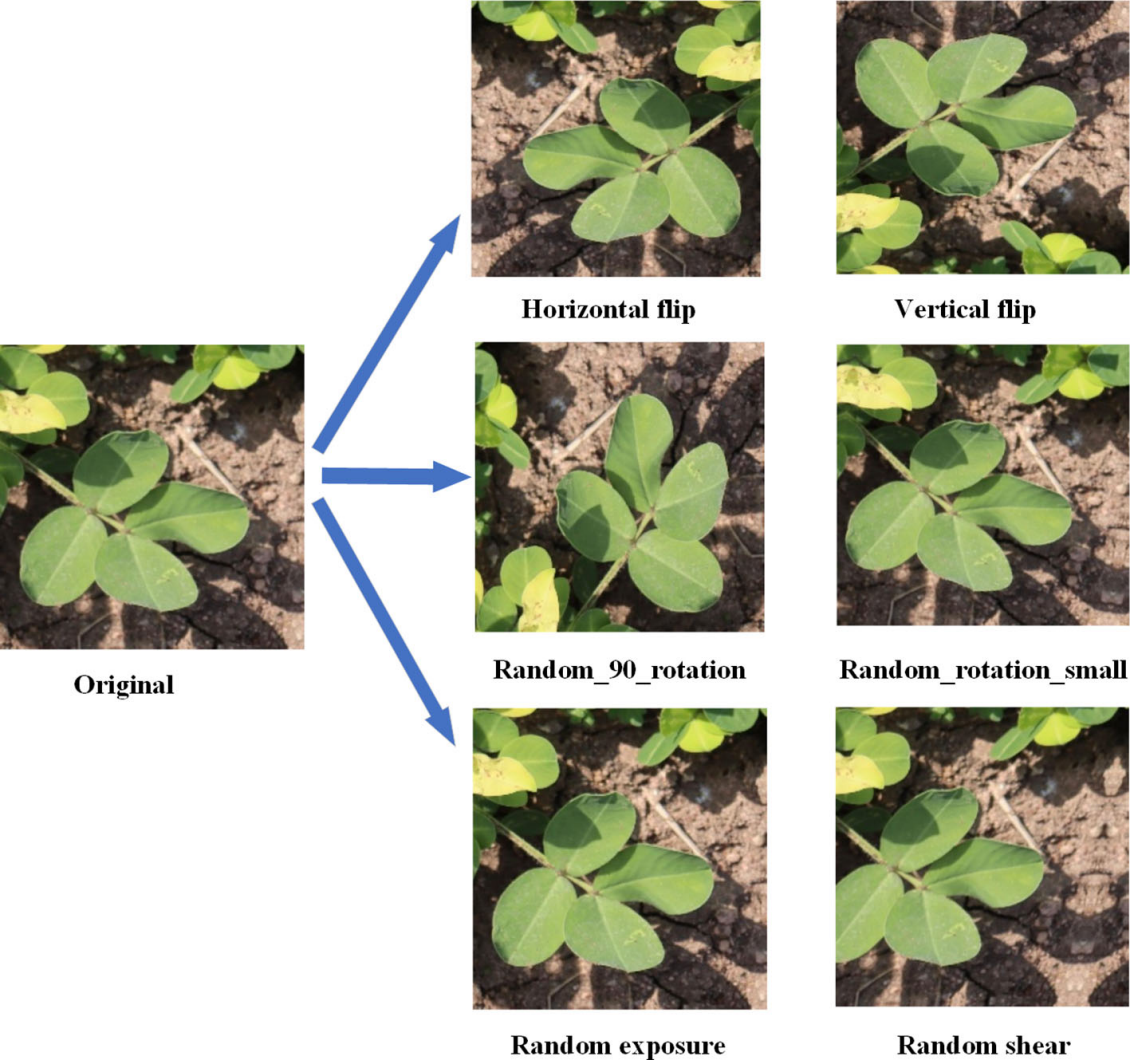
Data augmentation example diagram.

Considering the limited number of original images and the complex, variable nature of field environments, training models directly with raw data may lead to overfitting and poor generalization to broader application scenarios. To enhance the model’s robustness and generalization ability, we applied multiple data augmentation techniques to 2,132 annotated images, including: horizontal and vertical flipping (50% probability), 90-degree rotation (randomly selected from no rotation, clockwise rotation, counterclockwise rotation, or vertical flipping), brightness and contrast perturbation (randomly adjusting the original brightness by ±5% to 10%), and random cropping (randomly cropping in horizontal and vertical directions within a range of -15° to +15°). Examples of these augmentations are shown in **Figure 2**. These techniques effectively simulate diverse target appearances under varying poses, lighting conditions, and occlusions, thereby increasing the diversity of training samples. After augmentation, the total number of images expanded to 7,363. The dataset was then split into training, validation, and test sets in an 8:1:1 ratio, using a stratified random sampling strategy to ensure consistent class distribution and representativeness. Detailed information about the augmented dataset is provided in **Table 2**.

**Table 2:** Sample distribution after data augmentation.

Type	Number of images	Number of labels					
		0	1	2	3	4	5
Training set	5890	1020	990	980	975	970	955
Validation set	736	128	125	122	120	120	121
Test set	737	129	126	123	119	122	118
Total	7363	1277	1241	1225	1214	1212	1194

### 2.3 YOLO11 object detection model

YOLO11 (Khanam and Hussain, 2024), released by the Ultralytics team in 2024, represents another major breakthrough in the YOLO (You Only Look Once) series, particularly in terms of architectural design and performance optimization. Building on the end-to-end, single-stage detection framework advantages inherent to the YOLO series, this model further integrates multiple innovative structural modules, achieving a superior balance among detection accuracy, inference speed, and deployment flexibility. Depending on network depth and model complexity, YOLO11 is available in five variants—N, S, L, M, and X—to accommodate diverse application requirements ranging from resource-constrained edge devices to high-performance computing platforms. To evaluate the performance-efficiency trade-offs across these variants, we compared key metrics, including parameter count, mAP@50–95 accuracy, ONNX CPU and T4 TensorRT inference latency, and FLOPs, as detailed in **Table 3**.

**Table 3:** Performance comparison of five variants of the YOLO11 model.

Variant	Parameters (M)	mAP@50-95 (%)	FLOPs (G)	Inference speed ONNX CPU (ms)	Inference speed T4 TensorRT (ms)
YOLO11n	2.6	39.5	6.5	56.1 ± 0.8	1.5 ± 0.0
YOLO11s	9.4	47	21.5	90.0 ± 1.2	2.5 ± 0.0
YOLO11m	20.1	51.5	68	183.2 ± 2.0	4.7 ± 0.1
YOLO11l	43.7	53.4	86.9	238.6 ± 1.4	6.2 ± 0.1
YOLO11x	62.1	54.7	194.9	462.8 ± 6.7	11.3 ± 0.2

As shown in **Table 3**, the YOLO11n variant achieves reasonable detection accuracy (39.5% mAP) with the lowest parameter count (2.6M) and computational complexity (6.5G FLOPs), reducing the parameter count by approximately 24 times and FLOPs by over 30 times compared to the largest variant, X. This efficiency advantage makes it an ideal baseline model for resource-constrained scenarios.

And this efficiency advantage, in turn, stems from the several key architectural improvements that YOLO11 introduces to enhance feature representation and computational efficiency. First, the prediction head adopts the optimized C3k2 module, replacing the traditional C2f structure. This module uses two smaller convolutional kernels instead of a large one, reducing computational overhead and parameter count while allowing flexible switching between lightweight C2f and deeper C3 structures to meet diverse modeling requirements. Second, the Spatial Pyramid Pooling Fast (SPPF) (He et al., 2015) module is retained to enhance the receptive field, and a new C2PSA (Channel-to-Pixel Spatial Attention) mechanism is introduced to focus on critical spatial regions, improving detection performance for small and occluded targets.

Finally, the detection head employs Depthwise Convolution (DWConv) (Chollet, 2017) instead of standard convolution, further reducing computational complexity and making it well-suited for deployment in resource-constrained environments.

### 2.4 Improved model YOLO-PLnet

**Figure 3:**
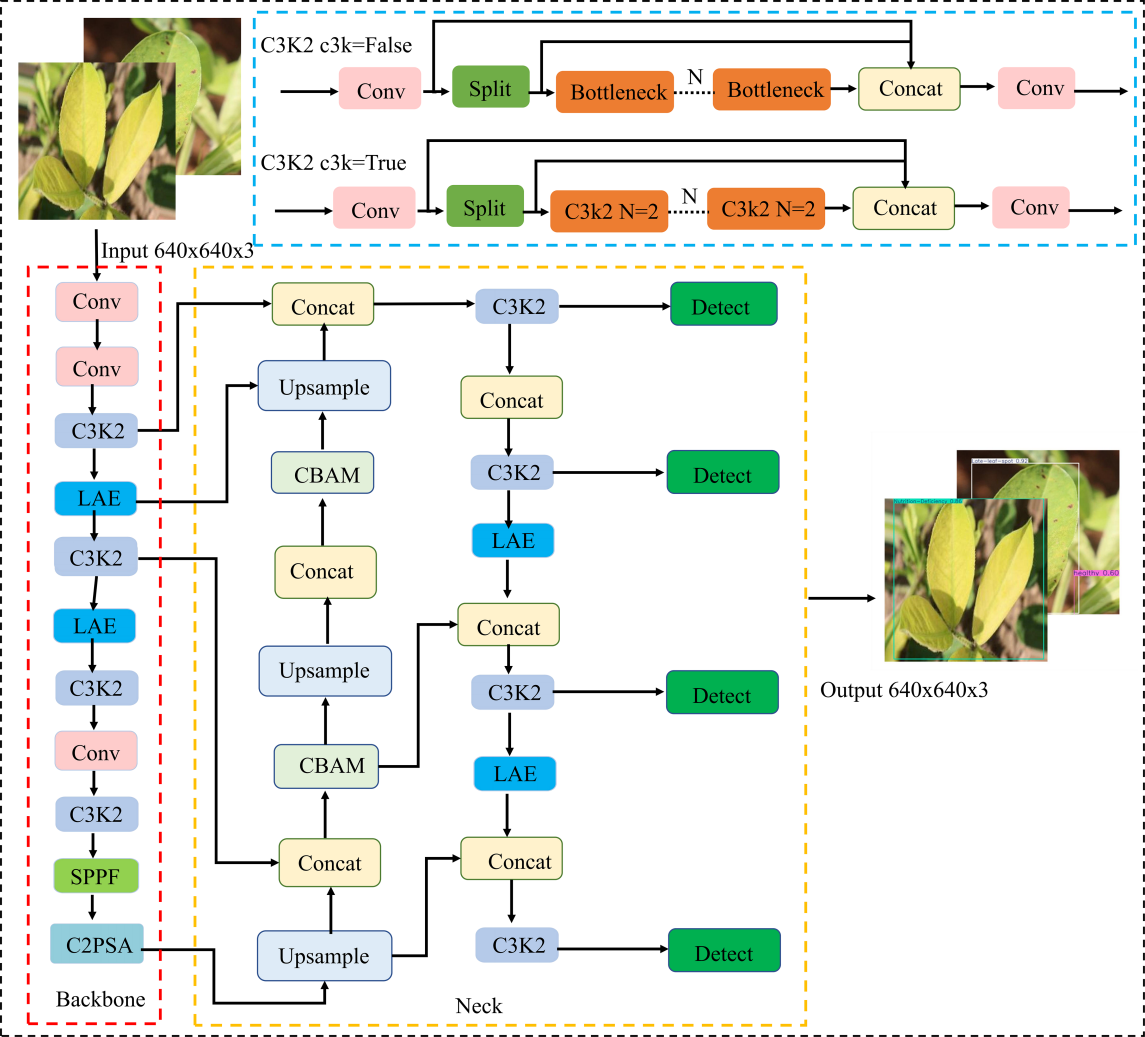
YOLO-PLnet model architecture.

To enable efficient deployment of a peanut leaf disease detection model on edge devices, lightweight improvements were made based on the YOLO11n model. First, the Lightweight Adaptive Feature Extraction (LAE) module was used to replace certain standard convolutions in the backbone and neck networks. This module achieves multi-scale feature fusion with lower computational complexity, enhancing the model’s perception of disease spot regions. Second, a Convolutional Block Attention Module (CBAM) was embedded in the multi-scale paths of the neck network to further strengthen feature responses in critical regions, improving the representation capability of high-resolution feature maps for small disease spots and targets with blurred edges. Additionally, the detection head was replaced with an Asymptotic Feature Pyramid Network (AFPN), which employs a staged cross-layer fusion strategy to effectively mitigate information inconsistencies caused by semantic differences, thereby improving the model’s adaptability and robustness in multi-scale disease detection tasks. The overall structure of the improved lightweight model, named YOLO-PLNet, is shown in **Figure 3**.

#### 2.4.1 LAE module

Traditional convolution operations, due to their local perception characteristics, tend to lose high-frequency information in image edges and corners during downsampling. These regions contain rich fine-grained features critical for object detection tasks. In peanut leaf disease detection, disease spots often appear at leaf edges or tips, and if edge information is weakened during downsampling, it can lead to inaccurate target responses, degrading the model’s detection performance. Moreover, standard convolutions fail to fully exploit information differences between adjacent pixels, often overlooking regions with high information entropy, which reduces the discriminability of feature representations.

**Figure 4:**
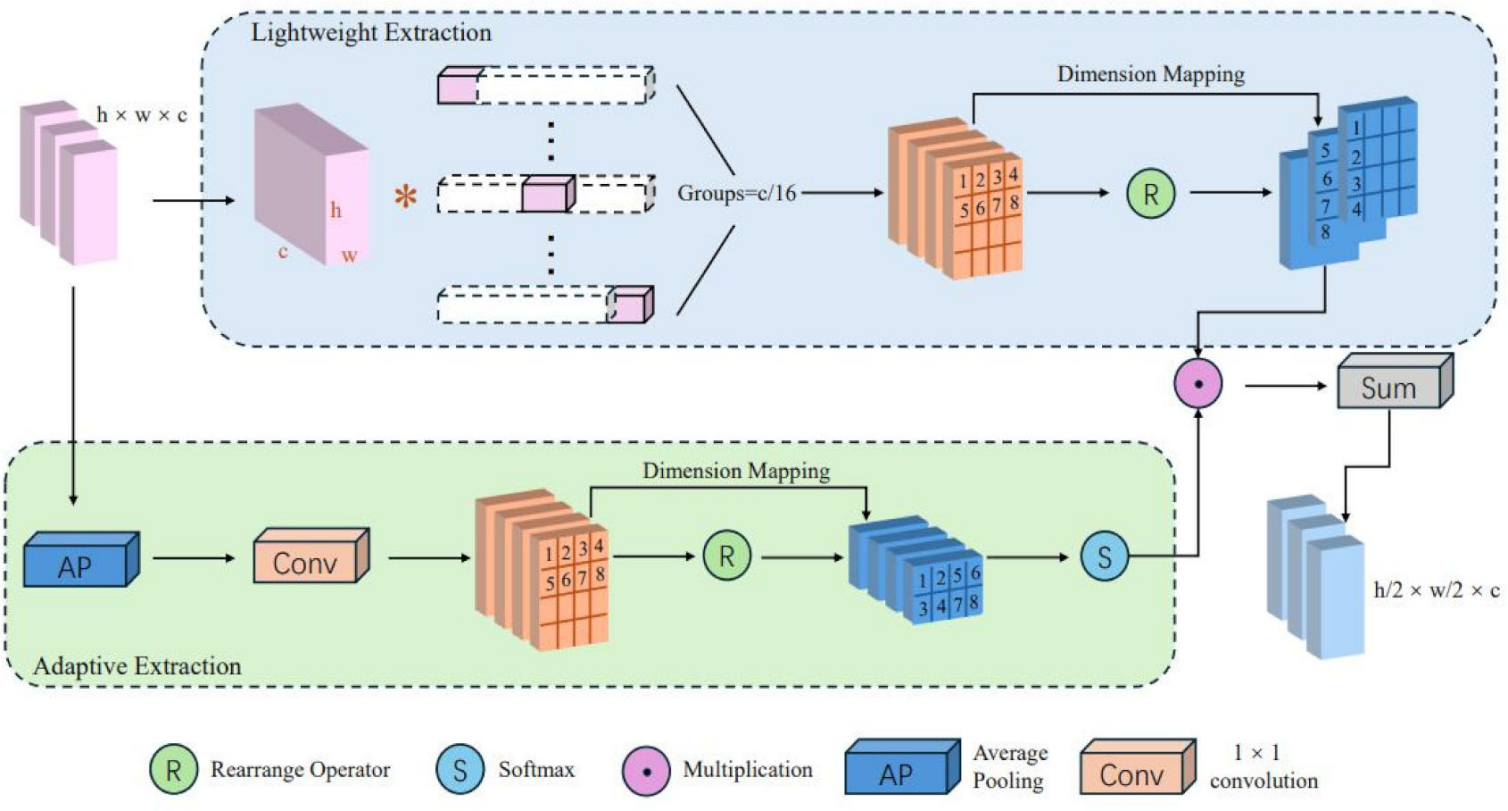
Module architecture of LAE.

The Lightweight Adaptive Extraction (LAE) module (Yin et al., 2024) employs a dual-branch structure, utilizing group convolution and an adaptive weighting mechanism to construct parallel paths, thereby enhancing the representation of edge details and target regions. The structure of the LAE module is illustrated in **Figure 4**. Compared to standard convolution, the LAE module reduces the parameter count to 1/N of the original and compresses spatial information into the channel dimension, thereby lowering computational complexity. The specific design of the LAE module is detailed as follows:

First, the input feature map $X\in {\mathbb{R}}^{B\times C\times H\times W}$is processed by group convolution (GroupConv), in which the channel dimension is divided into 16 groups ( G=C/16). Each group performs an independent 3×3 onvolution operation to further enhance the local feature representation capability. The process can be formulated as Equation 1:

(1) $$X^{\prime }={\text{GroupConv}}_{3\times 3}(X;G=\frac{C}{16})\in {\mathbb{R}}^{H\times W\times C}$$

Then, a dimension rearrangement operation converts the feature map into ${\mathbb{R}}^{H\times W\times C\times 4}$, forming four parallel multi-scale branches. A Channel Attention Mechanism (CAM) is applied to optimize the feature representation through global contextual recalibration. Specifically, a Global Average Pooling aggregates spatial information to generate global contextual descriptors, followed by a Softmax normalization to obtain the attention weights A. The process can be formulated as Equation 2:

(2) $$A=\text{Softmax}\left({\text{Conv}}_{1\times 1}\left({\text{AvgPool}}_{3\times 3}(X)\right)\right)$$

Finally, the attended feature Y is obtained by a weighted summation of all branches as Equation 3:

(3) $$Y=\sum_{i=1}^{4}{X^{\prime }}_{i}\cdot A_{i}\in {\mathbb{R}}^{H\times W\times C}$$

where ${X^{\prime }}_{i}$ and $A_{i}$ denote the feature map and the corresponding attention weight of the i-th branch, respectively. For the final detection head, a Depthwise Separable Convolution (DSConv) is applied for spatial down-sampling, which reduces the spatial resolution from H×Wto H/2×W/2while keeping the channel dimension unchanged. The operation can be expressed as Equation 4.

(4) $$\text{Output}=\text{DSConv}(Y)\in {\mathbb{R}}^{(H/2)\times (W/2)\times C}$$

#### 2.4.2 CBAM module

The self-attention mechanism originated in natural language processing (NLP) (Tsirmpas et al., 2024) and has found widespread application in computer vision. By establishing global dependencies between feature positions, it effectively enhances a model’s ability to capture contextual information, making it particularly suitable for visual tasks with complex spatial structures.

Considering the computational efficiency requirements of lightweight detection models, this study opted for the Convolutional Block Attention Module (CBAM) (Woo et al., 2018)—a structurally simpler and more adaptable alternative to the computationally intensive global self-attention mechanism. Specifically, CBAM is integrated into the Path Aggregation Network (PAN) multi-scale pathways of the YOLO-PLnet neck, positioned after the Concat and Upsample operations, targeting the fusion stage of multi-scale feature maps (P3, P4, P5). This placement enables precise optimization of channel dependencies and spatial attention foci during feature aggregation, enhancing the high-resolution feature maps’ ability to detect small targets while minimizing global computational overhead, aligning with the design goals of a lightweight model. To optimize CBAM performance, we employed Optuna for automated hyperparameter tuning, focusing on adjusting parameters such as the reduction ratio (channel compression rate), learning rate, and weight decay.

**Figure 5:**
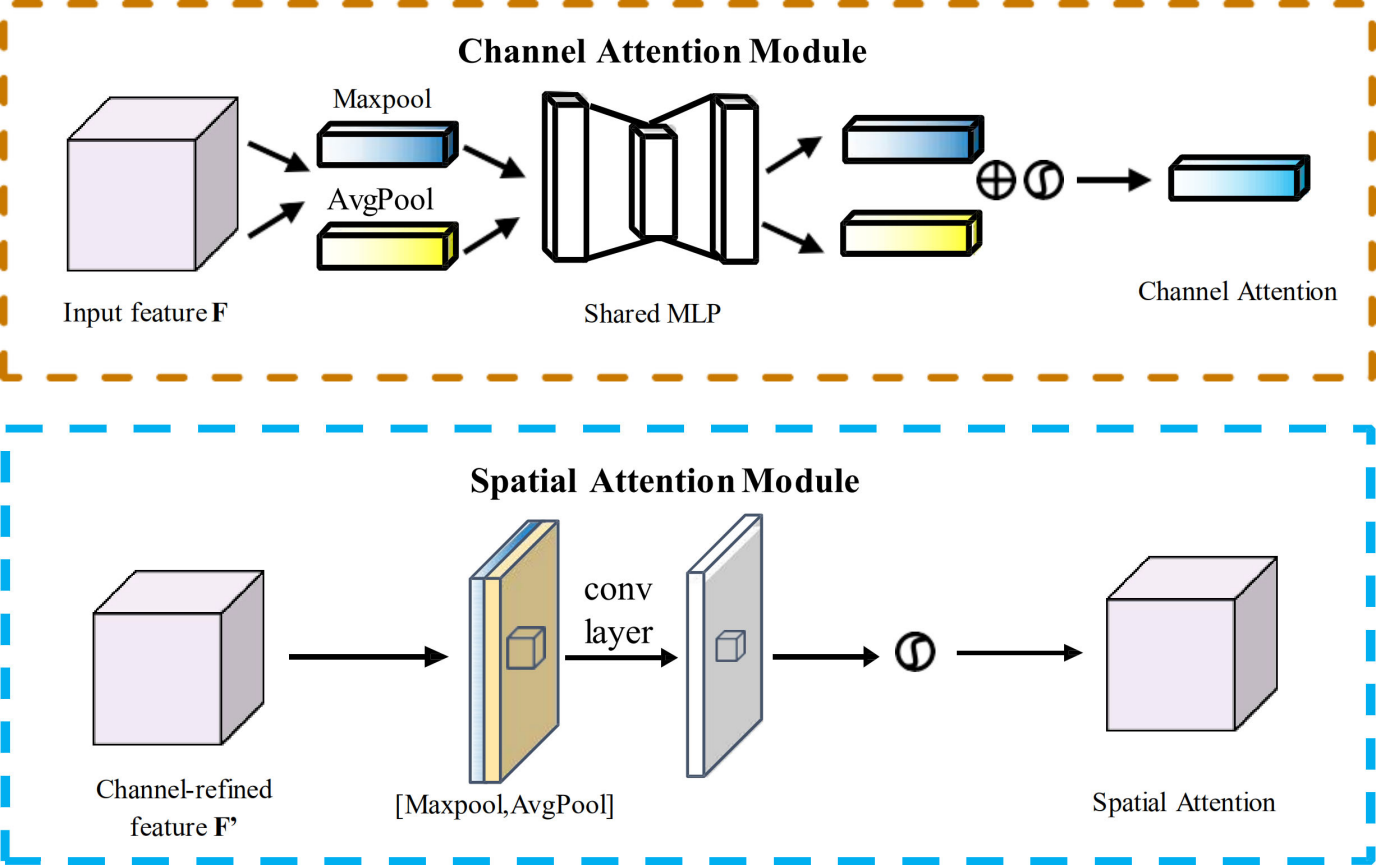
Diagram of each attention sub-module.

The CBAM module consists of a Channel Attention Module and a Spatial Attention Module (SAM) connected in series, with its model structure illustrated **Figure 5**. The computational processes for the CAM and SAM modules are described by Equations 5 and 6, respectively, while the feature fusion process is represented by Equation 7.

(5) $$M_{c}(F)=\sigma \left(\text{MLP}(\text{AvgPool}(F))+\text{MLP}(\text{MaxPool}(F))\right)$$

(6) $$M_{s}(F)=\sigma \left(f^{7\times 7}\left(\left[\text{AvgPool}(F);\text{MaxPool}(F)\right]\right)\right)$$

(7) $$F^{\prime }=M_{c}(F)⊗F,\;F^{\prime \prime }=M_{s}(F^{\prime })⊗F^{\prime }$$

Here, σ represents the Sigmoid activation function, and ⊗ denotes element-wise multiplication. Through the combination of these two-level attention mechanisms, CBAM effectively enhances the response strength of salient regions while suppressing redundant background information. Its lightweight structure makes it suitable for integration into lightweight detection models.

#### 2.4.3 AFPN module

**Figure 6:**
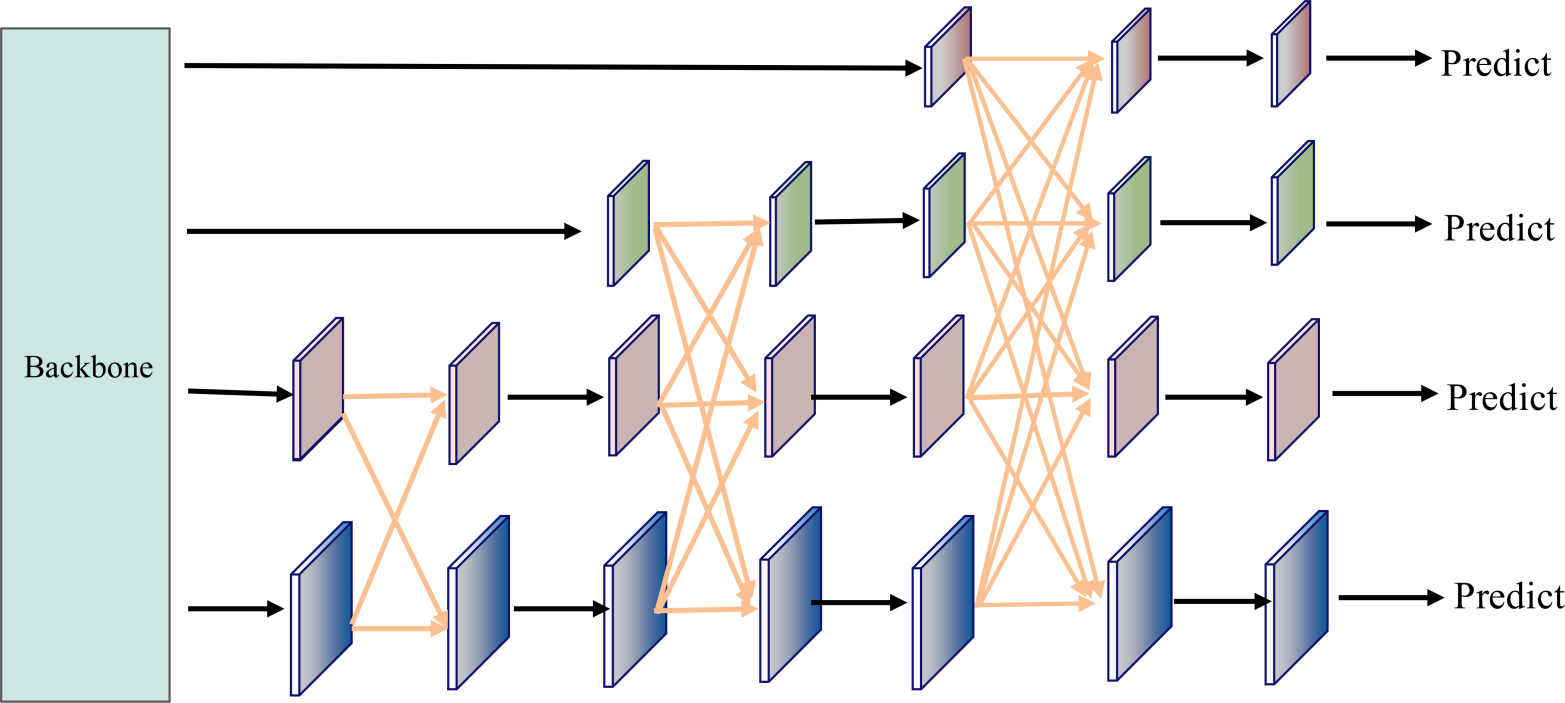
AFPN model architecture diagram.

To further enhance the detection performance for multi-scale disease spot regions, we integrated an Asymptotic Feature Pyramid Network (AFPN) into the original YOLO11n architecture, with its model structure depicted in **Figure 6**. AFPN employs a progressive feature fusion strategy from shallow to deep layers, aiming to mitigate the semantic misalignment issues caused by direct fusion of features at different scales in the traditional Feature Pyramid Network (FPN) (Yang et al., 2023). This module starts with adjacent shallow features (e.g., C2 and C3), gradually incorporating higher-level semantic features (e.g., C4 and C5), enabling layer-by-layer injection of cross-level information while maintaining semantic continuity. AFPN introduces lateral connections and cross-scale fusion paths between adjacent features across multiple stages, allowing low-level details and high-level semantics to align and collaborate effectively in a progressive manner.

Unlike the top-down information flow of traditional FPN, AFPN enables more flexible bidirectional communication and introduces low-level semantic information early through staged shallow prediction branches, enhancing the detection capability for small targets and edge regions. Additionally, the Adaptive Spatial Fusion (ASF) mechanism introduced by AFPN further strengthens the information coupling between multi-scale features. ASF dynamically allocates weights at each fusion stage based on the response intensity of feature positions, achieving finer spatial feature integration, effectively mitigating multi-scale information conflicts, and improving fusion consistency. This adaptive approach ensures robust performance across diverse field conditions. The integration of ASF also reduces computational redundancy, making it suitable for real-time applications. Furthermore, the enhanced feature alignment supports better generalization to unseen disease patterns. The cross-stage fusion process of AFPN is detailed in **Algorithm 1**.

**Algorithm 1:**
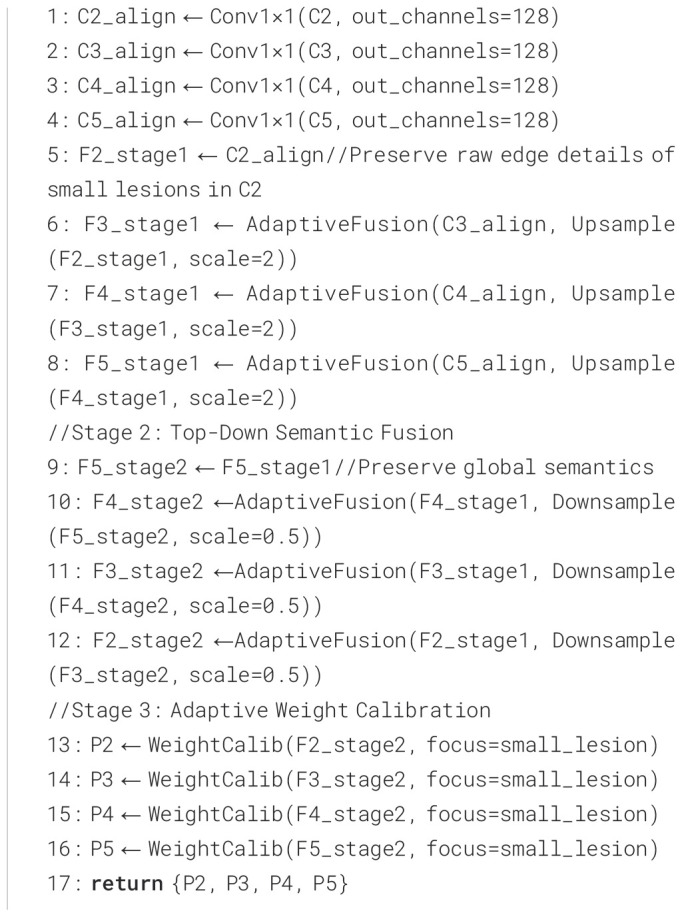
AFPN staged cross-level fusion.

### 2.5 Edge deployment platform

**Figure 7:**
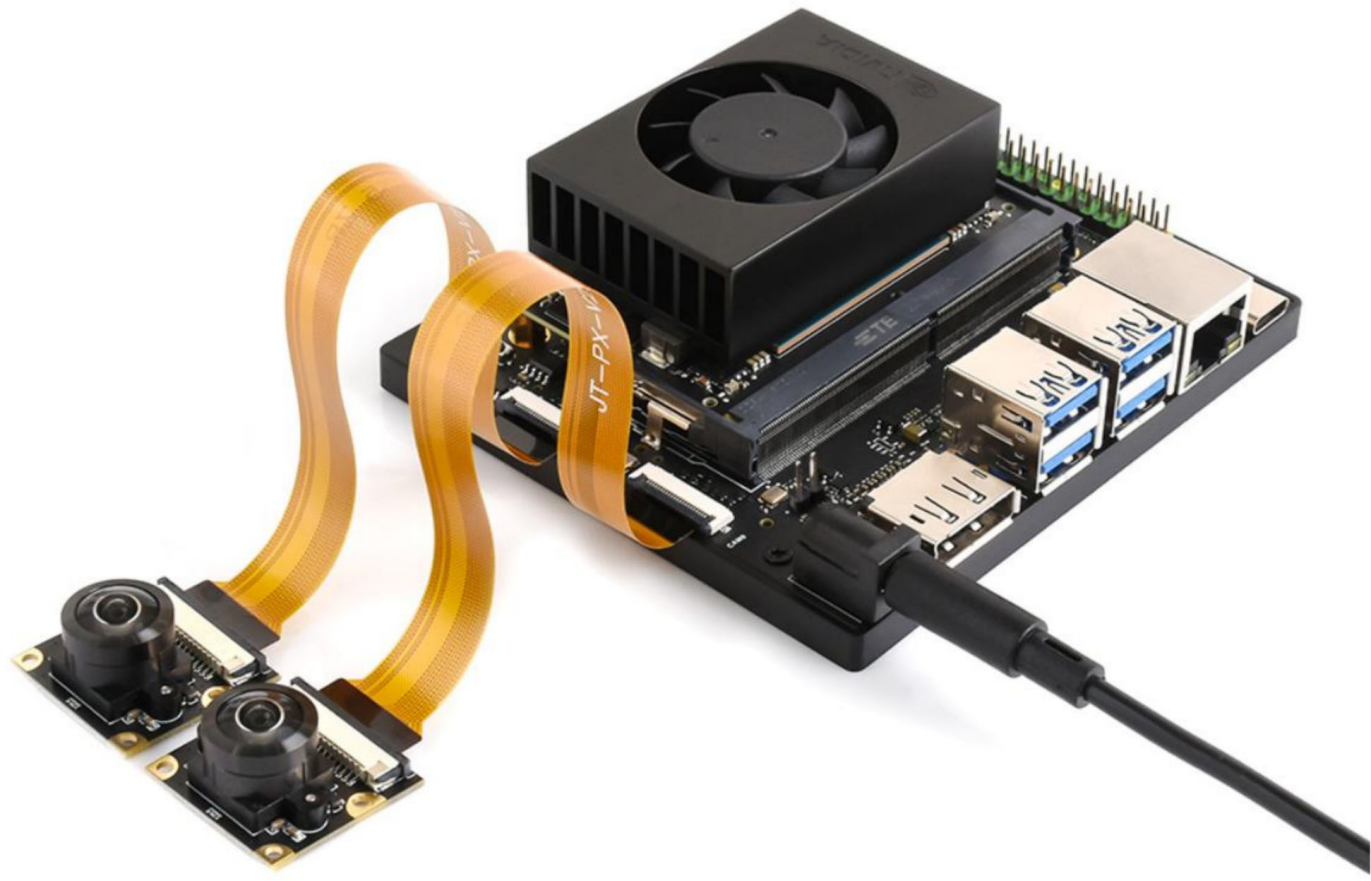
Jetson Orin NX developer kit for edge deployment.

To meet the requirements for real-time detection of peanut leaf diseases in field conditions, along with low latency and low power consumption, the study selected the NVIDIA Jetson Orin NX as the edge deployment platform. This device integrates 1,024 CUDA cores and 32 Tensor Cores, delivering an AI inference performance of up to 100 TOPS at INT8 precision. It can efficiently run deep neural networks locally, avoiding the delays and instability associated with network transmission. Additionally, the Jetson Orin NX supports the development of end-to-end accelerated AI applications through the JetPack SDK, facilitating model optimization, deployment, and on-site integration. As shown in **Figure 7**, this platform offers advantages such as miniaturization, high performance, and low power consumption, making it suitable for edge devices like agricultural robots and plant protection drones. This supports on-site localization and rapid response to peanut disease spots. Detailed hardware specifications are provided in **Table 4**.

**Table 4:** Jetson Orin NX parameters.

Parameter	Description
AI Performance	100 TOPS (INT8)
GPU	NVIDIA Ampere architecture with 1024 CUDA cores and 32 Tensor Cores
CPU	8-core Arm^®^ Cortex^®^-A78AE v8.2 64-bit, 2 MB L2 + 4 MB L3
Memory	16 GB 128-bit LPDDR5, 102.4 GB/s
Storage	Supports M.2 NVMe SSD
Video Encode	1× 4K60 (H.265), 3× 4K30 (H.265), 6× 1080p60 (H.265), 12× 1080p30 (H.265)
Video Decode	1× 8K30 (H.265), 2× 4K60 (H.265), 4× 4K30 (H.265), 9× 1080p60, 18× 1080p30 (H.265)
Power	Configurable 10W, 15W, 25W
Camera Interface	2× MIPI CSI-2 D-PHY camera connectors
USB	4× USB 3.2 Gen2 (Type-A), 1× USB Type-C (UFP)

## 3 Results

### 3.1 Experimental setup

To ensure the fairness and reproducibility of model training, all experiments were conducted on a unified laboratory server. The server operates on Ubuntu 20.04 LTS, with a configuration including a 16-core Intel(R) Xeon(R) Platinum 8352V CPU @ 2.10GHz, 120 GB of RAM, and an NVIDIA GeForce RTX 4090 (24 GB VRAM). The software environment consists of Python 3.8 + PyTorch 1.11, with CUDA version 11.3 and the cuDNN acceleration library integrated to enhance GPU inference efficiency. All models were trained on this platform using consistent hyperparameter settings to ensure result comparability, with training parameter configurations detailed in **Table 5**.

**Table 5:** Training parameter settings.

Hyperparameter	Value
Epochs	200
Image size	640 × 640
Batch size	16
Optimizer	SGD
Workers	16
lr0	0.005
lrf	0.01
momentum	0.937
Mosaic	False
Cross-Validation	5-fold stratified

### 3.2 Evaluation metrics

To comprehensively evaluate the model’s performance in object detection tasks, multiple evaluation metrics were used to assess the detection results, including Precision, Recall, and Mean Average Precision (mAP). Among these, mAP is a key metric for measuring detection accuracy and is widely used to evaluate a model’s ability to recognize targets across different IoU thresholds.

To assess the model’s robustness and generalization capability under varying detection conditions, two additional metrics, mAP@0.5 and mAP@0.5:0.95, were introduced. mAP@0.5 represents the average detection accuracy at an IoU threshold of 0.5, primarily measuring the model’s basic detection capability. mAP@0.5:0.95, on the other hand, calculates the average precision across IoU thresholds ranging from 0.5 to 0.95 (with a step size of 0.05), providing a more comprehensive reflection of the model’s detection stability and generalization performance across multi-scale and multi-class scenarios. The calculation formulas for the relevant evaluation metrics are shown in Equations 8-11.

(8) $$\text{Precision}=\frac{TP}{TP+FP}$$

(9) $$\text{Recall}=\frac{TP}{TP+FN}$$

(10) $$\text{AP}=\int_{0}^{1}P(R)dR$$

(11) $$\text{mAP}=\frac{1}{N}\sum_{i=1}^{N}AP_{i}$$

Here, TP represents the number of samples correctly detected as positive, FP denotes the number of samples incorrectly detected as positive, FN indicates the number of positive samples missed, and $AP_{i}$ refers to the average precision for the i-th class.

In practical deployment, the model must not only achieve high detection accuracy but also ensure operational efficiency in resource-constrained scenarios, particularly in agricultural contexts like peanut leaf disease detection, where models are often deployed on edge devices such as agricultural robots or field stations. To address this, four additional lightweight evaluation metrics were introduced: parameter count (Params), computational complexity (GFLOPs), model size (Model Size), and average detection frames per second (FPS), to quantify the model’s resource consumption and deployment performance.

Among these, the parameter count reflects the structural complexity of the model and serves as a core indicator of model compression, with its calculation method shown in Equation 12. GFLOPs represent the number of floating-point operations performed per second, indicating the model’s inference efficiency and computational load, with the formula provided in Equation 13. Model size indicates the storage space occupied by the model, calculated as shown in Equation 14. FPS measures the model’s ability to process images per unit time, reflecting its real-time inference performance, with the formula given in Equation 15. To further characterize resource consumption during deployment, three additional metrics—inference latency (Latency), power consumption (Power Consumption), and memory usage (Memory Usage)—were introduced. These metrics respectively reflect the time required for the model to process a single frame on edge devices, the level of energy consumption, and the utilization of memory resources.

(12) $$\text{Params}=O(\sum_{i=1}^{n}M_{i}^{2}\cdot K_{i}^{2}\cdot C_{i-1}\cdot C_{i})$$

(13) $$\text{GFLOPs}=O(\sum_{i=1}^{n}K_{i}^{2}\cdot C_{i-1}^{2}\cdot C_{i}+\sum_{i=1}^{n}M^{2}\cdot C_{i})$$

(14) $$\text{Model Size (MB)}=\frac{\text{Params}\cdot b}{8\cdot 10^{6}}$$

(15) $$\text{FPS}=\frac{N_{p}}{T}$$

Among them, $M_{i}$ represents the total number of samples correctly detected as positive in the i-th class; $C_{i}$denotes the total number of positive samples in the i-th class, with $C_{i-1}$ representing the cumulative sum of positive samples up to the (i−1)-th class; $N_{p}$ refers to the total number of positive samples across all classes; b and indicate the batch size (set to 32).

### 3.3 YOLO-PLnet performance detection

**Figure 8:**
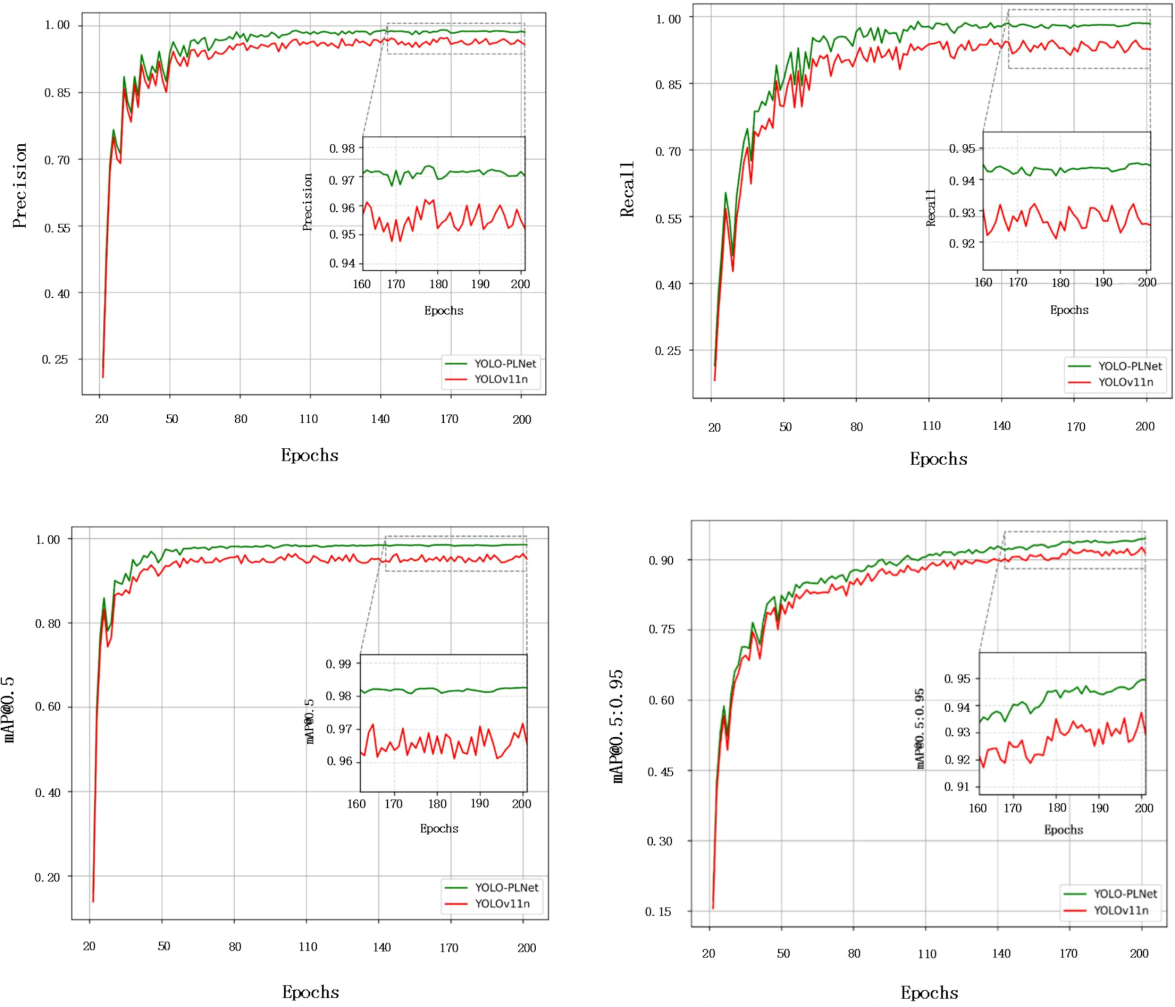
Performance comparison curves of YOLO-PLNet and YOLO11n.

To comprehensively compare the detection performance of the improved model with the baseline model, **Figure 8** illustrates the comparison of Precision, Recall, mAP@0.5, and mAP@0.5:0.95 for YOLO-PLNet and YOLO11n during the training process. It can be observed that within the first 110 epochs, the Precision and Recall curves of YOLO-PLNet still exhibit some fluctuations, reflecting the model’s adaptation and adjustment process to multi-scale target features in the early stages. As training progresses, the curves gradually stabilize and eventually converge. The final Precision and Recall reach 97.1% and 94.2%, respectively, representing improvements of 1.8% and 1.7% over YOLOv11n. Regarding accuracy metrics, mAP@0.5 stabilizes after the 80th epoch, while mAP@0.5:0.95 fluctuates and converges around the 155th epoch, ultimately achieving 98.1% and 94.7%, respectively.

The improvements in the aforementioned detection results are primarily attributed to the lightweight enhancements made to the baseline model in YOLO-PLNet. By introducing the LAE module with its parallel dual-branch convolution structure, the model enhances feature response capabilities across different scales and spatial distributions, enabling robust target perception even when dealing with unevenly distributed and blurred boundary disease spots on peanut leaves. This is particularly evident in disease types such as leaf spot and rust, where edge transitions are fuzzy and color changes are subtle; the module significantly elevates the model’s fine-grained discrimination level. Additionally, the integrated CBAM attention mechanism in the feature fusion paths effectively suppresses non-target interferences in complex field backgrounds, such as water stains and reflective spots, further strengthening the model’s focus distribution and representational stability on disease areas. The cross-scale information fusion strategy based on AFPN ensures higher representational consistency when integrating shallow texture features with deep semantic features, enhancing the model’s generalization capability for multi-scale disease regions on peanut leaves. This makes it well-suited for practical detection needs in natural field scenarios characterized by strong lighting variations, cluttered backgrounds, and unclear disease spot boundaries.

**Figure 9:**
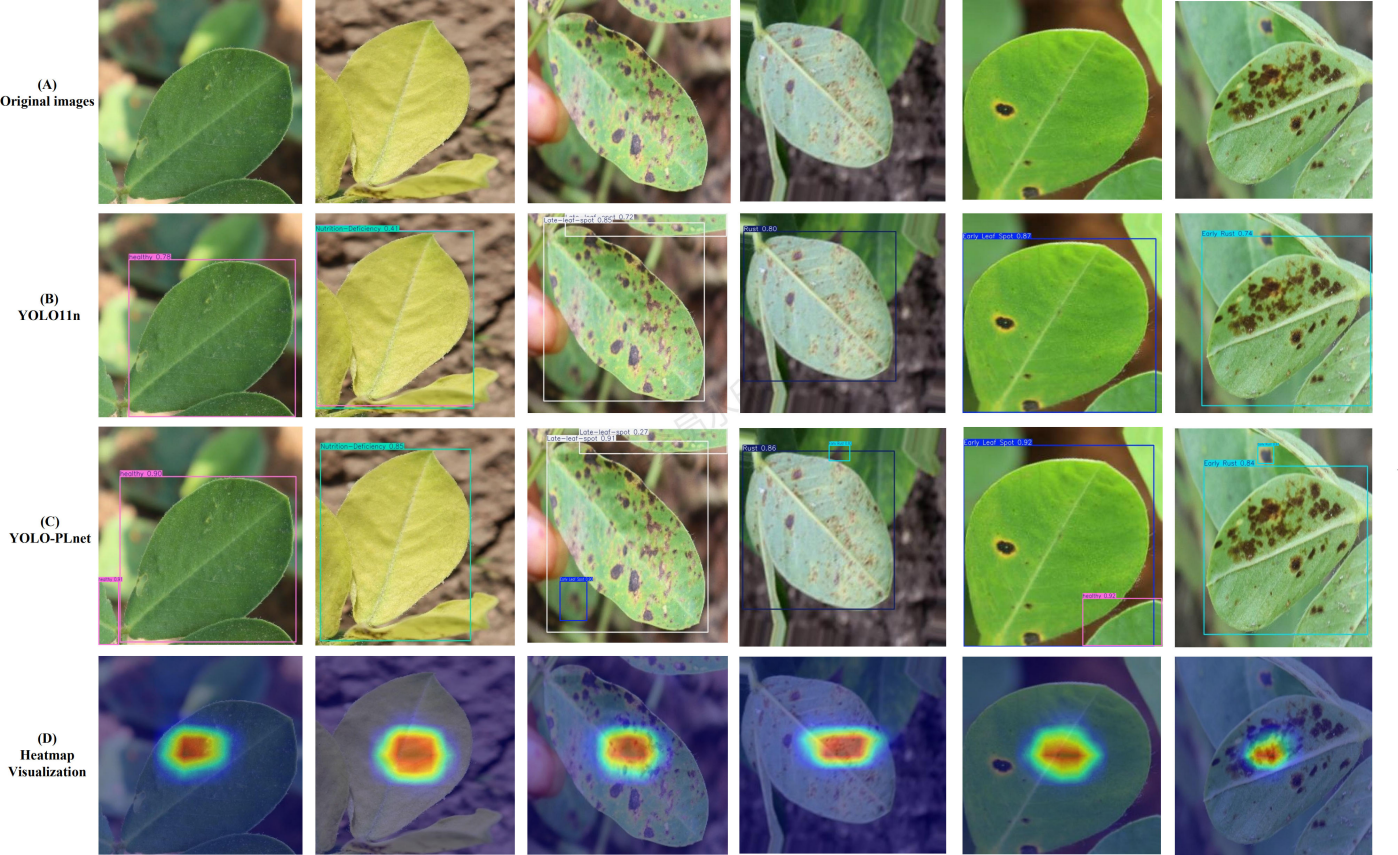
Visualization comparison of YOLO11n and YOLO-PLNet on peanut leaf disease detection.

**Figure 9** presents a visual comparison of the improved model YOLO-PLNet and the baseline model YOLO11n in the task of peanut leaf disease detection. Overall, YOLO-PLNet significantly outperforms the original model in terms of target localization accuracy and semantic focus capability. In the visualization of detection boxes, YOLO-PLNet more accurately covers disease spot regions, maintaining stable and reliable detection performance even in samples with densely distributed spots, blurred edges, or faint-colored mild lesions. In contrast, the YOLOv11n model exhibits issues such as missed detections, detection box offsets, and inaccurate edge judgments in some samples, indicating limitations in its perceptual ability under complex disease representations. To further analyze the model’s focus area distribution during inference, the study employed the Grad-CAM (Selvaraju et al., 2017) method to visualize the intermediate features of the YOLO-PLNet model. The heatmap results, shown in **Figure 9d**, clearly reveal that the activation regions are highly concentrated on the core areas of the disease spots, exhibiting a distinct hotspot response distribution. This demonstrates that the model can effectively extract discriminative features in complex backgrounds while maintaining stable attention on key regions.

**Figure 10:**
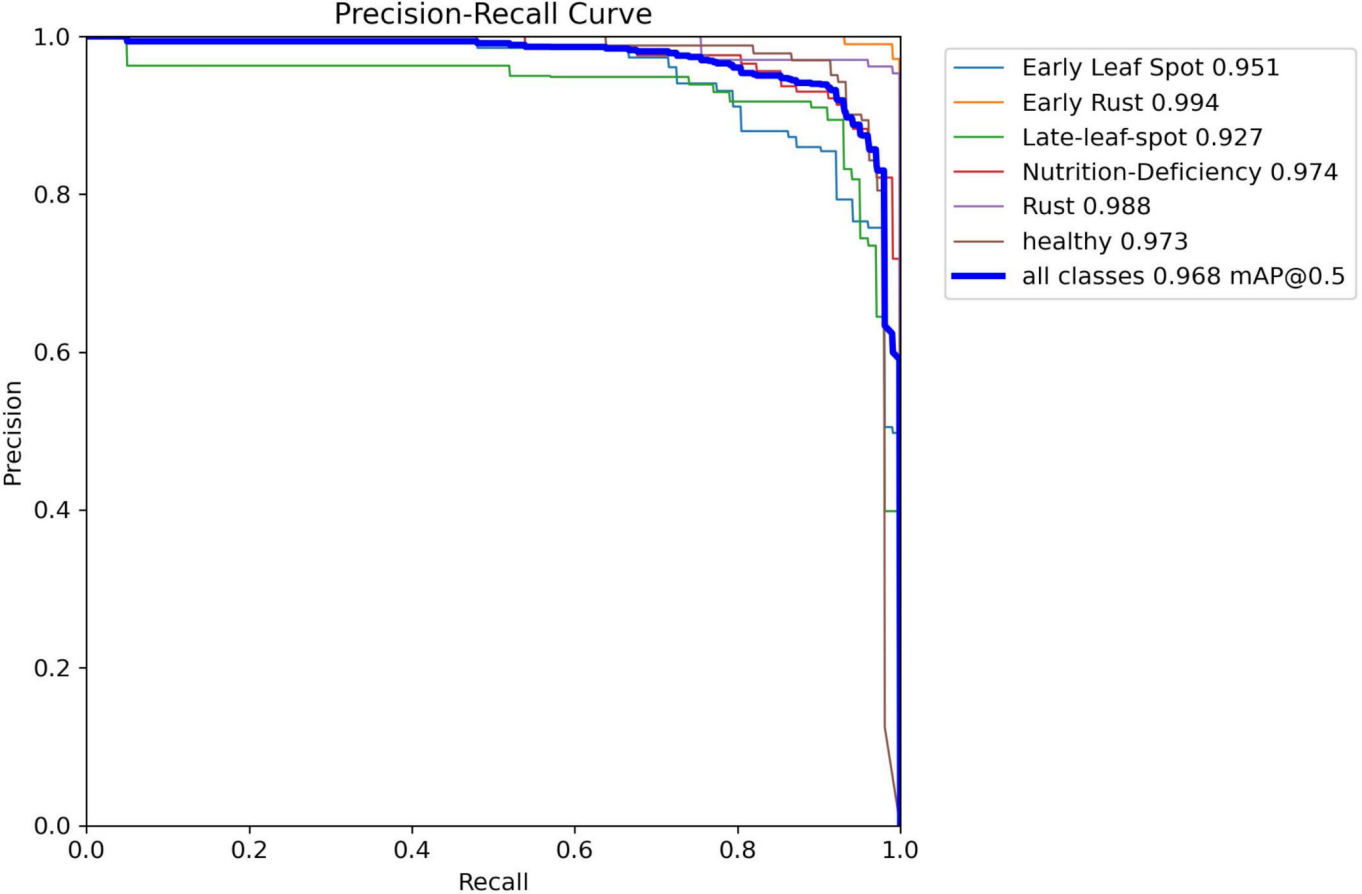
Precision-recall curve for peanut leaf disease detection.

Since peanut leaf diseases in the field typically exhibit a “one leaf, one disease” or “single-point outbreak” characteristic, a single image often contains only 1–2 disease targets, resulting in a relatively low number of bounding boxes in the detection results. However, during the data construction phase, class balance was carefully considered to ensure an even distribution of the six classes across the training and test sets (as detailed in **Table 2**), preventing model training bias. As shown in **Figure 10**, analysis via the Precision-Recall Curve reveals that YOLO-PLNet performs excellently across most categories. Notably, in the Early Rust category, the model achieves an AP of 0.994, nearly perfect, indicating extremely accurate target recognition for this class in images. In contrast, the AP for the Late-Leaf Spot category is 0.927, suggesting some challenges in recognizing this class, possibly due to similar disease spot features or complex backgrounds that make differentiation difficult for the model. Overall, YOLO-PLNet demonstrates stable and reliable comprehensive performance, achieving excellent AP results across most categories.

**Figure 11:**
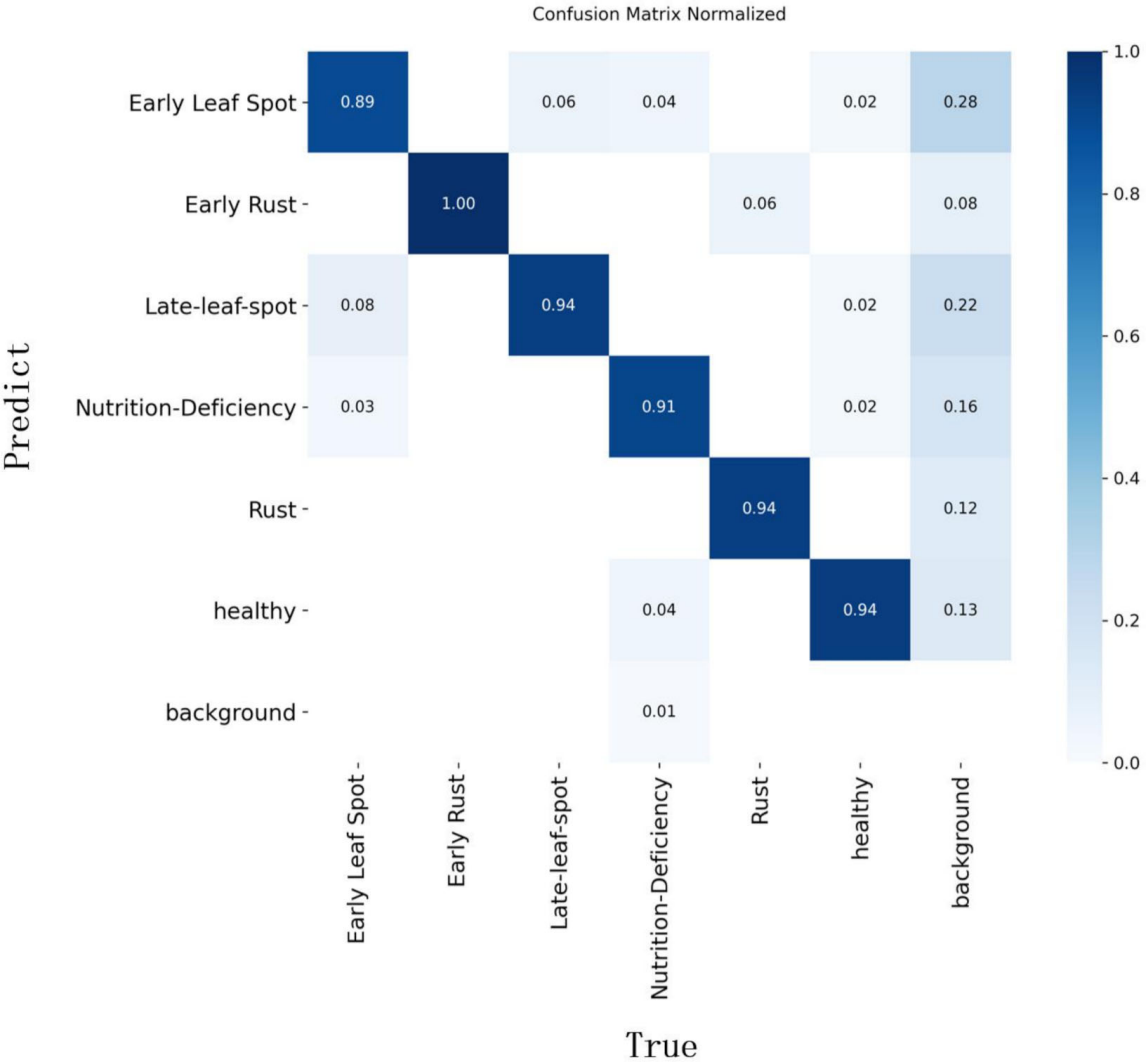
Confusion matrix of YOLO-PLNet on the test set.

To further analyze the detection performance and distinguishability of the YOLO-PLNet model across the six classes of peanut leaf disease targets, **Figure 11** presents the normalized confusion matrix for YOLO-PLNet on the test set. As shown in the figure, the prediction results for the Early Rust (1.00), Late-Leaf-Spot (0.94), Rust (0.94), and Healthy (0.94) categories are highly accurate, indicating strong stability in the model’s recognition of mainstream disease spots and healthy leaves. In contrast, the Early Leaf Spot (0.89) and Nutrition-Deficiency (0.91) categories exhibit some degree of confusion, with certain samples being misclassified as other disease categories or background. This may be attributed to similar disease spot features or background interference. Notably, the Early Leaf Spot category has a background misclassification rate as high as 28%, suggesting that the blurred boundaries or colors resembling the background of this disease type pose challenges for the model during recognition.

### 3.4 Lightweight performance comparison

To further evaluate the comprehensive performance of YOLO-PLNet in terms of accuracy retention and adaptability to edge deployment, this study conducted a comparative analysis with current mainstream lightweight detection models, including NanoDet-Plus (Sun and Zhuang, 2025), PP-YOLOE-s (Wang et al., 2025b), and RT-DETRv2 (Lv et al., 2024). As shown in **Table 6**, YOLO-PLNet achieves a superior balance among model complexity, inference speed, and detection accuracy.

**Table 6:** Comparison of YOLO-PLNet and lightweight detectors on edge.

Model	mAP@0.5 (%)	Params (M)	FLOPs (G)	Size (MB)	Inference Time(ms)
NanoDet-Plus	89.7	1.18	1.5	2.3	8.2
PP-YOLOE-s	93.1	7.9	18.2	15.6	18.4
RT-DETRv2	89.6	16.5	32	58	28
YOLOv8n	94.8	3.6	7.2	5.6	22.6
YOLO11n	96.5	2.6	6.5	5.35	21.3
YOLO-PLNet	**98.1**	**2.13**	**5.4**	**4.51**	**15.6**

Bold values indicate the results of the proposed YOLO-PLNet detector.

Experimental results demonstrate that YOLO-PLNet achieves excellent detection accuracy (mAP@0.5: 98.1%) while maintaining extremely low complexity (2.13 M parameters, 4.51 MB model size), showcasing a remarkable balance between precision and efficiency. Compared to similar lightweight models, YOLO11n and YOLOv8n, its detection accuracy improved by 1.6% and 3.3%, respectively. In deployment tests on the Jetson Orin NX platform using TensorRT-FP16, the inference latency was reduced by approximately 27% compared to the baseline model.

Further analysis from the perspective of inference efficiency reveals varying performance tendencies among the compared models on edge devices. NanoDet-Plus achieves the lowest inference latency (8.2 ms), but its detection accuracy is significantly lower, failing to meet the accuracy requirements of practical applications. PP-YOLOE-s, YOLO11n, and YOLOv8n exhibit inference times of 18.4 ms, 21.3 ms, and 22.6 ms, respectively, all meeting the basic frame rate requirements for real-time detection, yet their overall performance remains inferior to YOLO-PLNet. Conversely, RT-DETRv2, due to its complex architecture, has the highest inference latency (28.0 ms), limiting its applicability in edge warning scenarios that emphasize rapid response.

### 3.5 Ablation experiment

To systematically assess the actual contribution of the improved modules to the overall model performance, this study designed an ablation experiment as outlined in **Table 7**. Using YOLO11n as the baseline model, the lightweight feature enhancement module LAE, attention module CBAM, and multi-scale fusion structure AFPN were gradually introduced, with a comparative analysis conducted on their individual and combined configurations. Evaluation metrics, including mAP@0.5, mAP@0.5:0.95, parameter count, computational complexity, and model size, were used to quantify the trade-offs of each component in terms of performance and resource overhead.

**Table 7:** Ablation experiments of different modules.

YOLO11	LAE	CBAM	AFPN	mAP @0.5	mAP @0.5:0.95	Params (M)	FLOPs (G)	Size (MB)
✓	**×**	**×**	**×**	96.70%	93.00%	2.6	6.5	5.35
✓	✓	**×**	**×**	97.50%	93.50%	2.52	6	5.23
✓	✓	✓	**×**	97.70%	93.90%	2.45	5.6	4.95
✓	✓	✓	✓	**98.10%**	**94.70%**	**2.13**	**5.4**	**4.51**

Bold values indicate the results of the proposed YOLO-PLNet detector.

From the perspective of detection performance, the baseline model YOLO11 achieves mAP@0.5 and mAP@0.5:0.95 values of 96.7% and 93.0%, respectively. After introducing the LAE module, mAP@0.5 increases to 97.5%, mAP@0.5:0.95 rises to 93.5%, and the parameter count and computational complexity decrease to 2.52M and 6.0G, respectively. This demonstrates that the LAE module can maintain effective information perception while significantly reducing parameter redundancy and computational burden, thereby improving the model’s overall computational efficiency. The subsequent addition of CBAM further boosts mAP@0.5 to 97.7% and mAP@0.5:0.95 to 93.9%, confirming that the attention mechanism enhances the model’s response to key disease spot regions and improves its recognition of complex backgrounds and fine-grained features. The introduction of AFPN in the detection head, through adaptive weighting, enables dynamic fusion of multi-scale features, enhancing the model’s ability to detect disease targets of varying scales—especially small-scale spots—while effectively suppressing interference from low-quality features and maintaining low computational overhead. When all three modules (LAE, CBAM, and AFPN) are integrated simultaneously, YOLO-PLNet achieves optimal detection accuracy while effectively reducing model parameter count and weight size, providing strong support for the efficient detection of peanut leaf diseases and deployment on edge devices.

### 3.6 Comparison with baseline models

To further validate the comprehensive performance of the YOLO-PLNet model in object detection applications, a systematic comparison was conducted with eight representative detection models, including the classic two-stage detector (Faster R-CNN) (Ren et al., 2016), the lightweight single-stage detector SSD, and several mainstream lightweight versions of the YOLO series: YOLOv5n, YOLOv6n (Li et al., 2022), YOLOv7-tiny, YOLOv9t (Wang et al., 2025a), and YOLOv10n (Wang et al., 2024). The relevant results are presented in **Table 8**.

**Table 8:** Performance comparison of different object detection models.

Model	mAP@0.5 (%)	mAP@0.5:0.95 (%)	Params (M)	FLOPs (G)	Size (MB)
Faster R-CNN	55.7	20.3	40.1	34.7	339.7
SSD	51.2	29.4	29.51	30.1	169.72
YOLOv5n	84.3	79.2	3.23	7.6	7.81
YOLOv6n	88.4	82.7	4.82	12.2	8.32
YOLOv7-tiny	90.3	83.1	6.21	11.7	10.81
YOLOv9t	93.5	88.6	3.14	12.1	5.1
YOLOv10n	92.8	87.9	2.81	8.1	5.71
YOLO-PLNet (Ours)	**98.1**	**94.7**	**2.13**	**5.4**	**4.51**

Bold values indicate the results of the proposed YOLO-PLNet detector.

From the comparison results, the lightweight YOLO-PLNet model demonstrates clear advantages over mainstream object detectors in terms of detection accuracy and model complexity. YOLO-PLNet achieves an mAP@0.5 of 98.1% and an mAP@0.5:0.95 of 94.7%. Compared to the benchmark models YOLOv5n, YOLOv6n, YOLOv7-tiny, YOLOv9t, and YOLOv10n, YOLO-PLNet shows improvements in mAP@0.5 by 13.8%, 9.7%, 7.8%, 4.6%, and 5.3%, respectively, and in mAP@0.5:0.95 by 15.5%, 12.0%, 11.6%, 6.1%, and 6.8%, respectively. The largest improvement is 13.8% (relative to YOLOv5n), and the smallest is 5.3% (relative to YOLOv10n). Compared to the classic detectors SSD and Faster R-CNN, YOLO-PLNet also achieves significant enhancements in both accuracy metrics. In terms of lightweight metrics, YOLO-PLNet’s parameter count, FLOPs, and model file size are 2.13M, 5.4G, and 4.51MB, respectively, making it the best-performing among all compared models. This fully demonstrates that the improved YOLO-PLNet model offers high detection accuracy while providing effective support for real-time detection tasks in resource-constrained scenarios.

### 3.7 Edge deployment and inference evaluation

**Figure 12:**
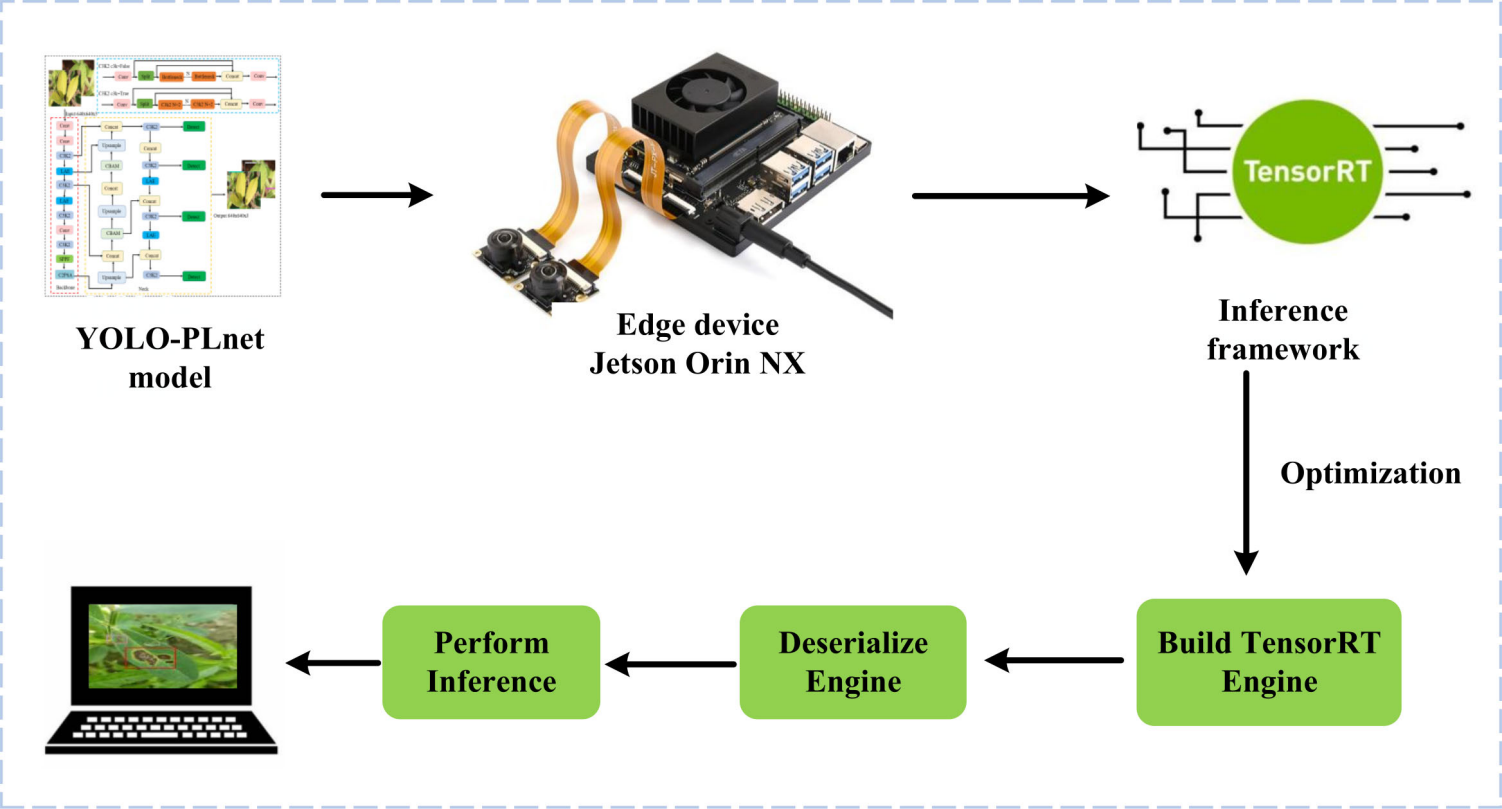
Edge deployment and inference process.

With the advancement of edge computing technology, the deployment of deep learning models not only prioritizes high accuracy but also requires balancing runtime speed and resource usage. To verify the deployment adaptability and real-time inference capability of the improved model YOLO-PLNet on edge devices, we established a complete inference pipeline covering real-world peanut field scenarios, as shown in **Figure 12**. This pipeline builds directly on the Jetson Orin NX Developer Kit (**Figure 7**), which serves as the hardware foundation.On the platform, the trained YOLO-PLNet model was first exported from the.pt format to an ONNX intermediate representation and then optimized into an.engine inference engine file using the TensorRT toolchain. To simulate the dynamic monitoring needs in the field, an industrial-grade CSI (Camera Serial Interface) camera was introduced, capable of capturing real video streams that include natural lighting variations and leaf movement interference. By deserializing and loading the.engine file with the TensorRT runtime, combined with the input of real field video streams from the camera, frame-by-frame real-time inference and visualization were performed, validating the model’s deployment performance in dynamic and complex edge scenarios.

To verify the inference efficiency of the model on edge devices, this study conducted experiments based on the edge deployment inference pipeline shown in **Figure 12**, utilizing the mainstream inference framework TensorRT (Xia et al., 2022). TensorRT offers various precision optimization methods, including FP16 half-precision and INT8 integer quantization deployment modes. The FP16 precision uses half-precision floating-point representation, offering significant advantages such as fast computation speed and low memory usage, making it widely used in efficient inference scenarios on edge devices. In contrast, INT8 precision quantizes weights and activation values into 8-bit integers, enabling further reduction in model size and device power consumption while maintaining a certain level of accuracy, which aligns well with the needs of resource-constrained edge devices.

After completing model training, the Post-Training Quantization (PTQ) (Li et al., 2021) toolchain provided by TensorRT was used to directly convert the original ONNX model (FP32 precision) into FP16 and INT8 precision versions, avoiding the high cost of retraining. These were then deployed on the Jetson Orin NX platform to verify the impact of different quantization strategies on deployment performance, with the detailed results presented in **Table 9**.

**Table 9:** Deployment performance comparison under FP16 and INT8 precision configurations.

Quantization Precision	Latency (ms/img)	FPS(frame/s)	GPU Memory Usage (%)	Power Consumption(W)	mAP@0.5 (%)
FP32	32.5	15.4	62.3	5.2	98.1
FP16	19.1	28.2	52.1	4.1	98
INT8	11.8	41.3	48.6	3.4	97.5

The results indicate that differences in detection accuracy under varying quantization precisions during edge deployment are minimal, while the improvements in inference efficiency and resource utilization are significant. The INT8 quantized model reduces inference latency by 38.21% and increases frame rate by 46.45% compared to FP16, while also featuring lower memory usage and power consumption. Compared to the original FP32 precision, the inference efficiency is substantially enhanced, directly supporting resource-constrained, real-time detection scenarios such as peanut field agricultural machinery patrols, particularly for battery-powered agricultural drones.

Although the YOLO-PLNet model was trained with a fixed input size of 640×640, the industrial CSI camera in actual deployment may differ from the model input size due to variations in field of view and video resolution. To assess the model’s adaptability to real-field data, tests were conducted with three input sizes: 320×320, 640×640, and 1280×1280, with the relevant results presented in **Table 10**.

**Table 10:** Deployment performance of YOLO-PLNet under different input sizes.

Input Resolution	Latency (ms/img)	FPS(frame/s)	Power Consumption(W)
320×320	16.7	52.4	3
640×640	19.1	28.2	4.1
1280×1280	32.6	13.4	6

The experimental results show that higher resolutions lead to increased inference latency and power consumption. While higher resolutions can retain more information about leaf disease spots, the focus of this experiment was to evaluate inference performance across multiple resolutions. After consideration, 640×640 was selected as the recommended input size. In actual deployment, the field video streams captured by the camera will be preprocessed to this size for input into the model, with inference results output directly to the agricultural machinery cab display via a visualization interface, providing operators with intuitive disease warnings.

**Figure 13:**
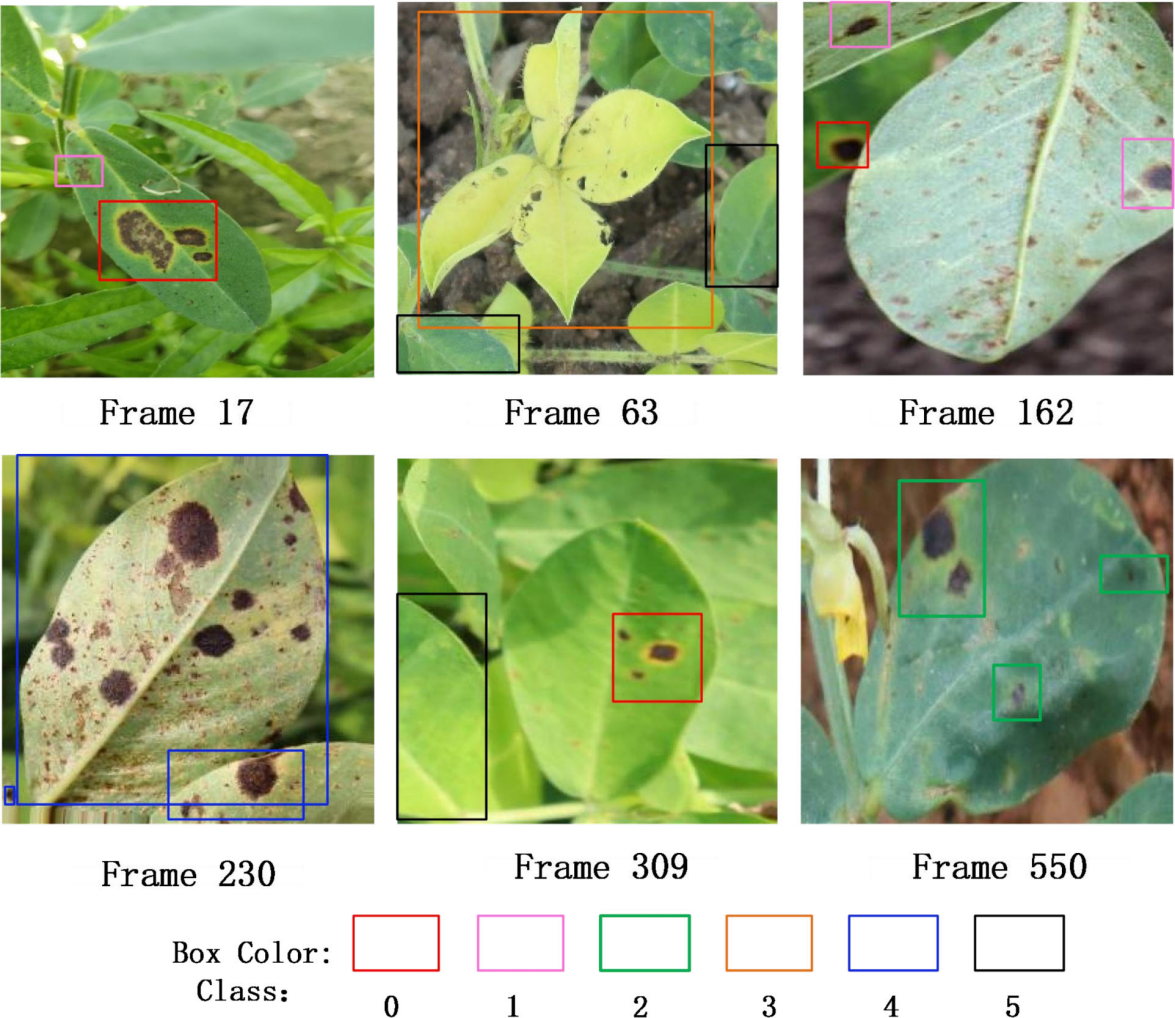
Detection results of YOLO-PLNet on key frames from video stream.

**Figure 13** displays the keyframe detection results obtained from video stream inference, covering six classes of peanut leaf diseases (categories 0–5), represented by bounding boxes in different colors. The inference process was conducted on the Jetson Orin NX platform, and the results visually demonstrate the model’s ability to detect and recognize real peanut leaf images in the field on edge devices, verifying its feasibility and practical value in actual deployment environments.

## 4 Discussion

In recent years, crop disease detection methods based on deep learning have emerged as a focal point in the research of intelligent agricultural monitoring (Khan et al., 2025). Numerous studies have attempted to enhance the identification capability and detection accuracy of disease spot regions by optimizing target detection network architectures. Liu et al. proposed the A-Net model, an improvement on YOLOv5, incorporating attention mechanisms, Wise-IoU loss function, and RepVGG modules, effectively boosting apple leaf disease detection accuracy to an mAP@0.5 of 92.7% (Liu and Li, 2024). Xie et al. developed a strawberry disease detection model based on YOLOv8, integrating CBAM, DySample, and ODConv, achieving an accuracy of 98.0% (Xie et al., 2024a). However, these approaches predominantly focus on improving algorithmic precision while overlooking various challenges in practical agricultural applications, particularly the limitations of computational resource constraints and high real-time requirements when deploying disease detection models on edge computing devices, which hinder their widespread adoption in real-world scenarios.

To address these challenges, this study proposes an improved target detection model, YOLO-PLNet, based on YOLO11n. One of the most significant findings is the substantial enhancement in the model’s ability to detect small disease spots through the introduction of the AFPN. In the early stages of peanut diseases, leaf spot and rust often manifest as tiny features spanning only a few pixels. Traditional feature pyramid networks tend to lose these details during multiple downsampling steps. In contrast, our fusion strategy, by reinforcing the shallow feature flow in the top-down pathway, effectively integrates high-resolution features rich in detail with low-resolution features rich in semantics. This enables the model to retain the overall context of the leaf while accurately identifying and locating these minute early-stage spots, thereby providing a technical foundation for early warning and intervention.

Furthermore, accurately classifying visually similar diseases is crucial. For peanut leaf spot and rust, their distinct biophysical characteristics are key: leaf spot manifests as dark necrotic patches with noticeable texture changes, while rust is characterized by yellowish-brown urediniospore pustules and distinct spectral features, such as a reflection peak at 520–600 nm (Lin et al., 2025). YOLO-PLNet integrates lightweight channel and spatial attention mechanisms, enabling adaptive calibration of feature maps. For instance, when processing suspected rust areas, it enhances responses to specific color channels; when analyzing leaf spot, it prioritizes edge and texture variations. This targeted learning of disease-specific biophysical features aligns closely with recent research using attention mechanisms to decode subtle plant phenotypic patterns (Tang et al., 2025). Comparative evaluations demonstrate that YOLO-PLNet achieves mAP@0.5 of 98.1% and mAP@0.5:0.95 of 94.7%, surpassing existing benchmarks. Its edge deployment on the Jetson Orin NX further validates its practicality. At INT8 precision, it delivers a real-time detection speed of 41.3 FPS, offering a critical advantage for resource-constrained platforms. Although INT8 quantization results in a slight accuracy drop, the resulting reductions in power consumption and speed improvements are vital for battery-powered mobile platforms, such as agricultural drones and inspection robots. This efficiency is supported by the Orin NX’s approximately 100 TOPS INT8 performance, enabling uninterrupted field operations and meeting the growing demand for sustainable edge AI in agriculture.

Although this study constructed a high-quality peanut leaf disease dataset and developed a lightweight detection model tailored for edge devices, certain limitations remain. First, the model’s generalization ability requires further improvement. Its performance has not been fully validated across diverse regional peanut varieties, unknown disease types, or cross-crop scenarios (e.g., corn, soybean), which may limit its adaptability in varied agricultural environments. Second, there are potential risks related to data privacy and commercial confidentiality. The current centralized training approach, when deployed across multiple farms, may lead to the leakage of sensitive agricultural information, such as planting layouts and variety characteristics, posing ethical and security concerns.

To address these limitations, future research can pursue the following directions: First, to enhance cross-scenario adaptability, advanced techniques such as domain adaptation and open-set recognition should be explored to improve the model’s compatibility with diverse crops, unknown diseases, and complex field conditions. Second, a privacy-preserving collaborative training framework should be developed, leveraging federated learning to enable distributed training across farms, integrating multi-source farm data for iterative optimization.

## 5 Conclusion

This study proposes a lightweight object detection model for peanut leaf disease, YOLO-PLNet, designed for edge deployment. Built on the foundation of YOLOv11, the model incorporates structural improvements such as LAE, CBAM, and AFPN to enhance feature representation capability and detection accuracy.

The main research conclusions are as follows:

1. The YOLO-PLNet model achieved an mAP@0.5 of 98.1% and an mAP@0.5:0.95 of 94.7% on the peanut disease dataset. Additionally, its parameter count, FLOPs, and model size were reduced by 18.07%, 16.92%, and 15.70%, respectively, compared to the baseline model YOLOv11n, effectively achieving model lightweighting and providing favorable conditions for edge deployment.
2. Deployed on the Jetson Orin NX edge device, the INT8 precision quantization, compared to FP16, reduced inference latency by 38.21%, increased FPS by 46.45%, and significantly lowered memory usage and power consumption, while detection accuracy slightly decreased to 97.5%. This enables the model to perform efficient real-time disease detection on low-power embedded platforms.
3. Under multi-resolution inputs, 640×640 delivered optimal inference results on the edge device, making it suitable for moderately resolved image streams in farmland scenarios. Combined with CSI camera video input, it enabled end-side automatic detection and visualized feedback.

In summary, the proposed YOLO-PLNet model offers high accuracy, low resource consumption, and excellent engineering deployability, making it suitable for peanut leaf disease monitoring tasks on agricultural edge intelligent terminals. In the context of sustainable agricultural development, this model can provide farmers with precise disease warning information, enabling targeted disease management, reducing unnecessary pesticide use, and promoting green agriculture. Future research could integrate the model with IoT sensors to further enhance its real-time performance and adaptability in practical agricultural scenarios, contributing to the widespread adoption of agricultural automation and intelligent management.

## Data Availability

The original contributions presented in the study are included in the article/supplementary material. Further inquiries can be directed to the corresponding author.
